# Induction of oxidative stress and cell apoptosis by selenium: the cure against oral carcinoma

**DOI:** 10.18632/oncotarget.22752

**Published:** 2017-11-29

**Authors:** Bin Qiao, Baoxia He, Jinghua Cai, Alfred King-Yin Lam, Wei He

**Affiliations:** ^1^ Department of Stomatology, The First Affiliated Hospital of Zhengzhou University, Zhengzhou, Henan, P.R. China; ^2^ Department of Pharmacy, Affiliated Cancer Hospital of Zhengzhou University, Henan Cancer Hospital, Zhengzhou, Henan, P.R. China; ^3^ Cancer Molecular Pathology, School of Medicine and Menzies Health Institute Queensland, Griffith University, Gold Coast, Australia

**Keywords:** oral carcinoma, selenium, oxidative stress, apoptosis, cell proliferation

## Abstract

Oral carcinoma (OC) remains one of the most difficult malignancies to cure. selenium (Se) is an essential trace mineral for human and animals, but high concentrations of Se induce apoptosis and oxidative effects. Although cell apoptosis has been evidenced as a critical mechanism mediating the anticancer activity of Se, the underlying molecular mechanisms remain elusive. To explore the role of Se in rat OC, we examined the weather the oxidative stress-mediated apoptotic pathway induced by Se was involved in the development of OC. In this study, we successfully constructed the OC rat model by 4-Nitroquinoline-1-oxide (4-NQO) exposure which reflected from histopathological observations. Se-induced the productions of methane dicarboxylic aldehyde (MDA) and reactive oxygen species (ROS), which was accompanied by the inhibition of superoxide dismutase (SOD) both *in vivo* and vitro. The anti-apoptotic gene (Bcl-2) was down-regulated and pro-apoptosis members (Bax, Bak, Cyt-c, caspase9 and caspase3) were up-regulated by Se in OC cells. Meanwhile, we also found that Se could strongly inhibited the cell proliferation of OC lines *in vitro*. These results suggested that excessive Se could effectively cause oxidative stress and induce apoptosis in OC cells, as a result the OC was also inhibited to some extent. Therefore, the information presented in this study is believed to be helpful in supplementing data for further therapy of OC.

## INTRODUCTION

Cancer is the leading cause of death worldwide. Five percent of all cancer occur in the head and neck, and approximately half of those occur specifically in the oral cavity [1]. Oral carcinomas (OC) are the world’s eleventh most common form of human neoplasm and account for 3% of all newly diagnosed cancer cases [2–4]. Despite efforts to improve overall outcomes, survival rates have not changed during the last 20 years [5]. Tongue cancer is the most common oral cavity neoplasm with an unfavorable prognosis and high metastatic potential [6]. Patients with premalignant lesions and early stage cancers have a high rate of survival, but the vast majority of Stages III and IV cases are fatal [7]. Therefore, the study of oral cancer has become the focus of the majority of scholars.

The essential trace mineral, selenium (Se), is an essential micronutrient for humans and animals [8–10]. It was discovered by Berzelius in 1817 and was named after the moon goddess. Se is an essential chemopreventive antioxidant element to oxidative stress, although high concentrations of Se induce toxic and oxidative effects [11]. In various animal models, Se possesses a potent cancer chemopreventive activity when its intake exceeds the nutritional requirement by 10-fold [12]. Sodium selenite (Na_2_SeO_3_) is commonly used as a dietary supplement for the treatment of Se deficiency [13]. The chemopreventive intake of Se may exert protection against cancer development by inducing the loss of transformed epithelial cells [14]. Apoptosis, a mode of cell death, is a physiologic event that regulates cell number and eliminates damaged cells. Recent studies have implicated that apoptosis is a common mechanism through which chemotherapeutic agents exert their cytotoxicity and that the efficiency of anti-tumor agents is related to the intrinsic propensity of the target tumor cells to respond to these agents by apoptosis [14]. Furthermore, apoptosis genes can improve tumor chemosensitivity and treatment outcome [15]. But, whether the Se have therapeutic effectiveness on the OC, and whether it improved the OC via caspase-mediated apoptotic pathway with the alteration of intracellular redox state.

In this present study, to explored the possible role of caspases in OC cell apoptosis induced by Se, we examined the involvement of these molecules in the apoptosis of the rat OC cells induced by Se. We found Se could strongly induce oxidative stress via inhibiting the activities of superoxide dismutase (SOD), and up-regulating the contents of methane dicarboxylic aldehyde (MDA) and reactive oxygen species (ROS) in OC cells. Meanwhile, the caspase-apoptosis and the decreased proliferation was also induced by Se in rat OC model. The effects suggest that Se possesses a potent cancer chemopreventive activity via apoptotic pathway. These data will provide valuable clues regarding the therapy for OC.

## RESULTS

### The effects of selenium on antioxidative activities in OC tissues

**Figure 1:**
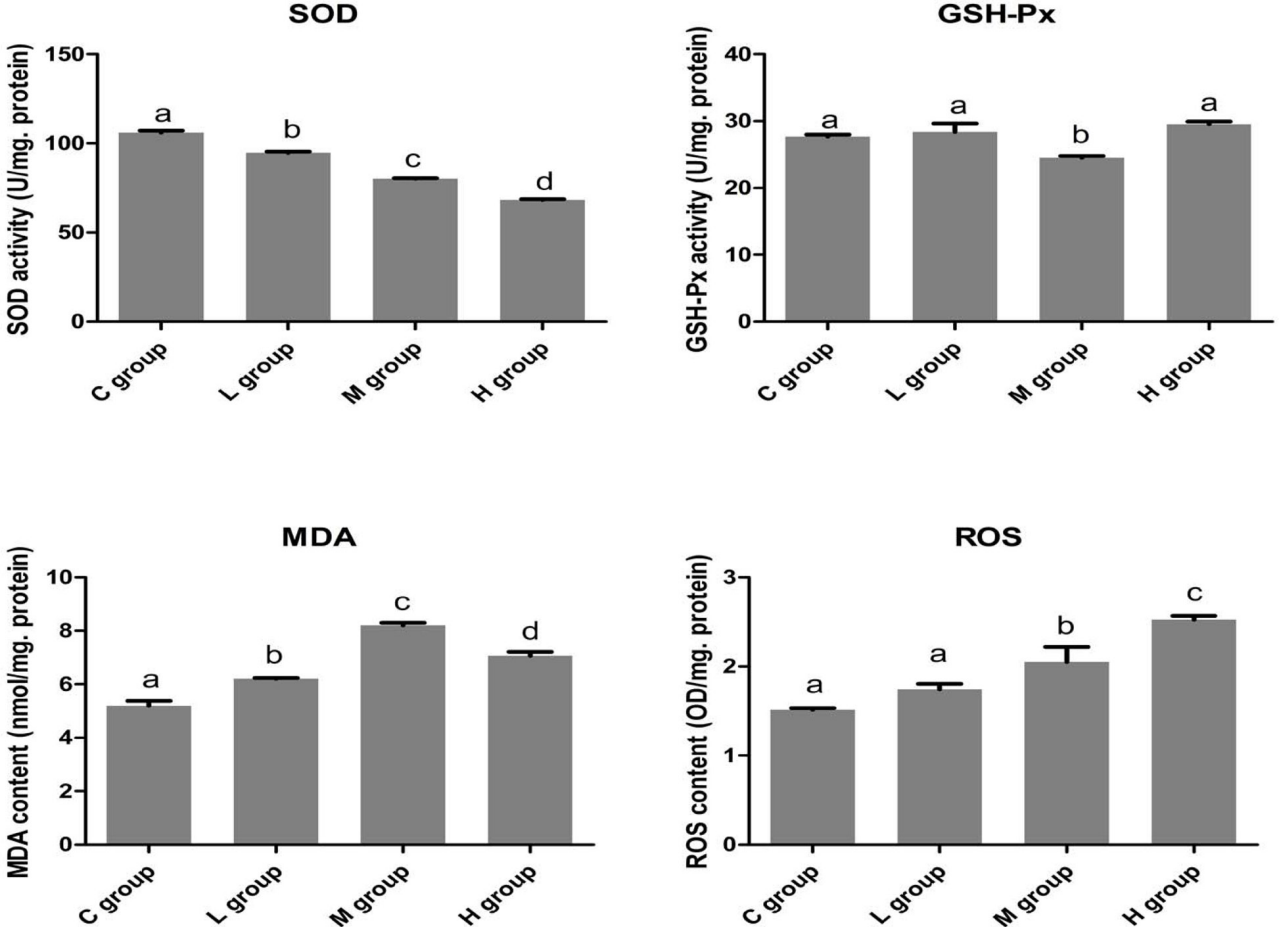
The effects of Se on oxidative stress of OC tissues The levels of SOD, GSH-Px, MDA and ROS was displayed. Bars with the different small letters represent significant differences (*P* < 0.05) between any two group. Each value represented the mean ± SD of five individuals.

To examine the effects of Se on redox state in OC tissues, we detected the activities of antioxidative enzymes (SOD and GSH-Px) and the contents of oxidative productions (MDA and ROS). The results are respectively summarized in Figure 1. In the Se treated-groups, the activities of SOD in OC cells decreased, but the activities of GSH-PX have no obvious changes with increasing concentrations of Se. In contrast, MDA and ROS levels increased with increasing concentrations of Se.

In the OC tissues (*n* = 5), the activities of SOD were significantly lower (*P* < 0.05) in all groups of OC tissues that were exposed to Se compared with C group. In H group, the SOD activity reached its lowest level and significantly decreased approximately by 35.58% compared with its C group.

The activetise of GSH-Px were down-regulated (*P* < 0.05) only in M group. But, the levels of MDA and ROS increased (*P* < 0.05) with the increasing concentration of Se in the OC tissues. Moreover, the contents of MDA and ROS reached their pinnacle in M group and H group, respectively. Therefore, these results showed that more Se could strongly induce oxidative stress in the OC tissues.

### The effects of Se on cell apoptosis in OC tissues

**Figure 2:**
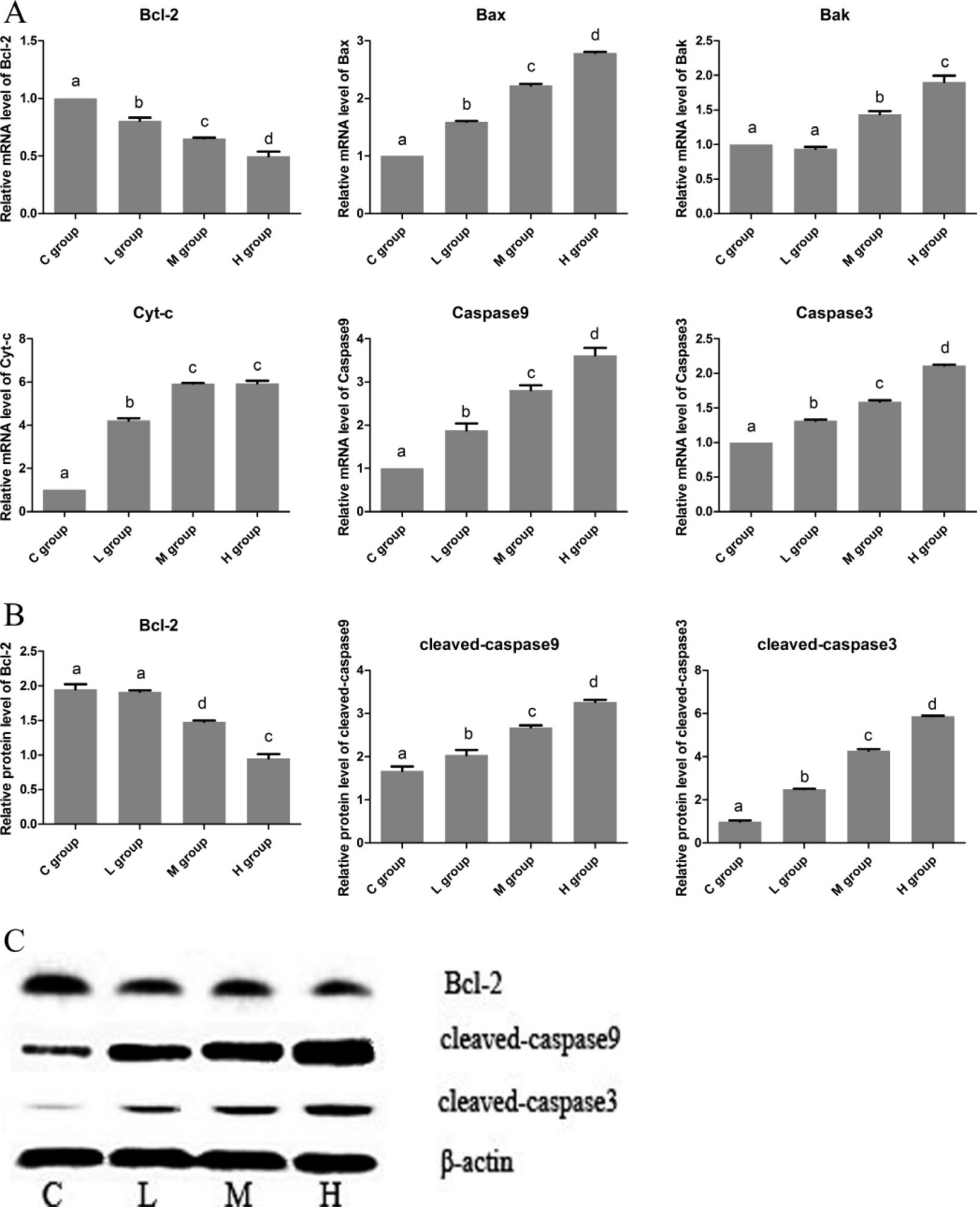
The effects of Se on cell apoptosis of OC tissues (**A**) is the mRNA levels of apoptosis gens (Bcl-2, Bax, Bak, Cyt-c, Caspase9 and Caspase3). (**B**) is protein levels of apoptosis gens (Bcl-2, cleaved-caspase9 and cleaved-caspase3). (**C**) is the immunoblotting of Bcl-2, cleaved-caspase9, cleaved-caspase3 and β-actin. Bars with the different small letters represent significant differences (*P* < 0.05) between any two group. Each value represented the mean ± SD of five individuals.

To identify the effect of Se on cell apoptosis of OC, the mRNA and protein levels of related apoptosis genes (Bcl-2, Bax, Bak, Cyt-c, caspase9 and caspase3) were measured. In Figure 2, we found both the mRMA and protein levels of anti-apoptotic gene (Bcl-2) were decreased with the increase of Se dose. Especially, the Bcl-2 expression was reached the lowest level, it decreased to 49.67% in H group when compared with C group. However, the levels of pro-apoptotic genes (Bax, Bak, Cyt-c, caspase9 and caspase3) showed significant increasing trends, and all the highest expression levels of pro-apoptotic genes appeared in H group.These results indicated that Se could induce cell apoptosis of OC in an dose manner.

### The effects of Se on OC cell proliferation

**Figure 3:**
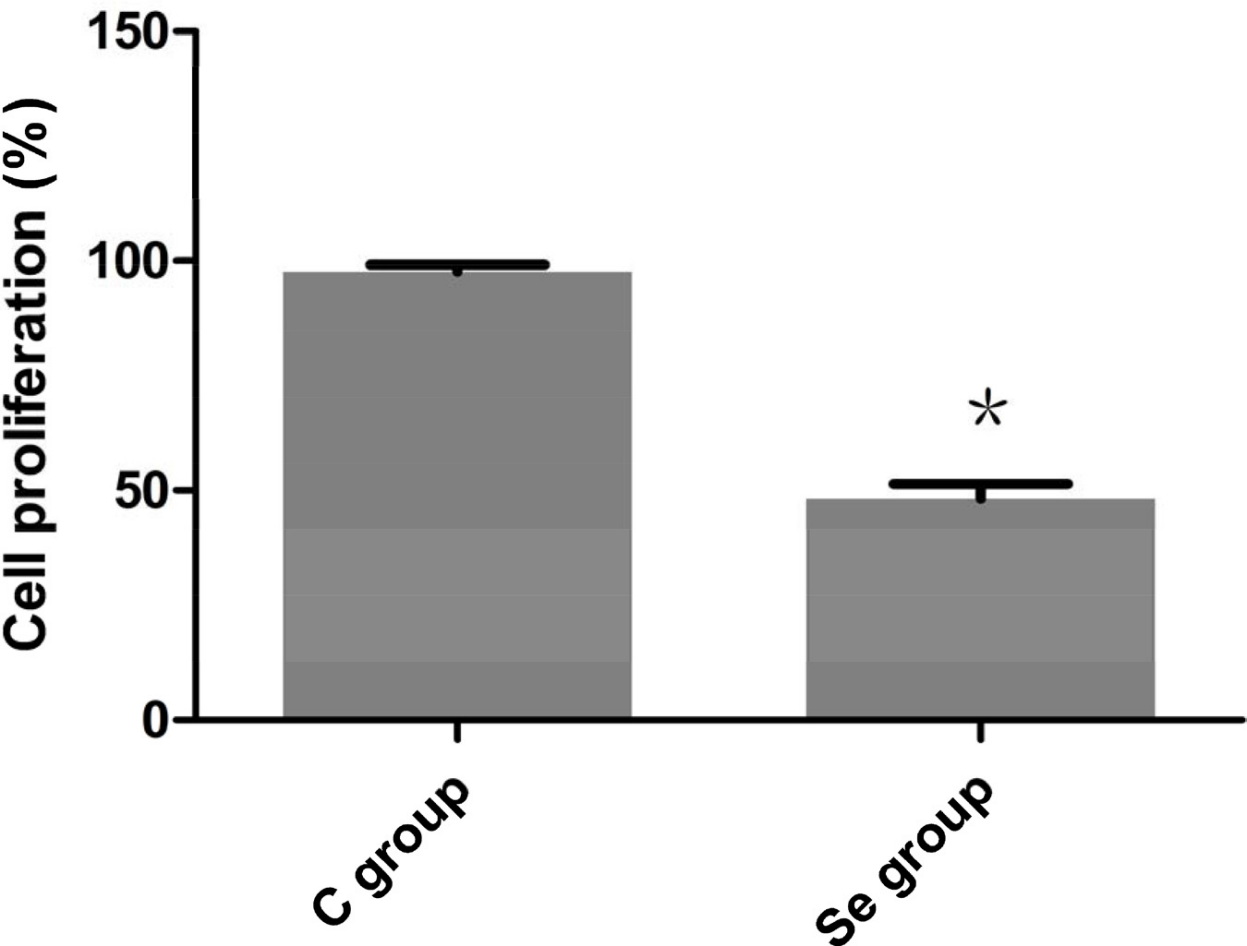
The effects of Se on proliferation of oral squamous carcinoma cells Asterisk represents significant differences (*P* < 0.05) between control group and Se-treated group. Each value represented the mean ± SD of five individuals.

To study the efficacy of Se on proliferation of OC cell, MTT assays was used in this work. As shown in Figure 3, compared with its corresponding C group, the cell proliferation of Se group was decreased by about 50% (*P* < 0.05%). This result suggested that Se strongly inhibited the growth of OC cells.

### The effects of Se on oxidative stress of oral squamous carcinoma cells

**Figure 4:**
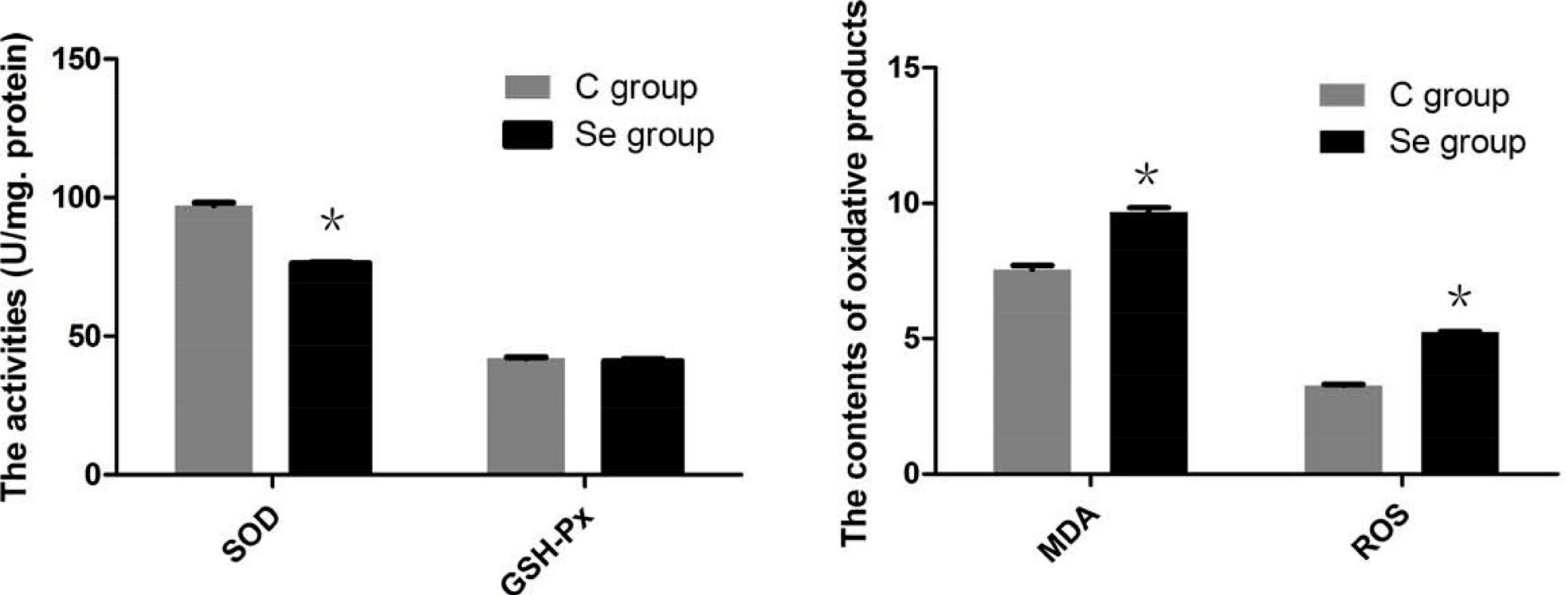
The effects of Se on levels of SOD, GSH-Px, MDA and ROS of oral squamous carcinoma cells Asterisk represents significant differences (*P* < 0.05) between control group and Se-treated groups. Each value represented the mean ± SD of five individuals.

To better understand the actions of Se on oxidation reaction, we detected the intracellular redox state by reagent kit. As shown in Figure 4, the antioxidant enzyme (SOD) was significantly decreased to 78.50% by treated Se in oral squamous carcinoma cells (*P* < 0.05), the activity of GSH-Px has no obvious changes (*P* > 0.05). But the contents of oxidation products (MDA and ROS) were up-regulated by Se in oral squamous carcinoma cells (*P* < 0.05). These results were consistent with the experiments of *in vivo*.

### The effects of Se on apoptosis of oral squamous carcinoma cells

**Figure 5:**
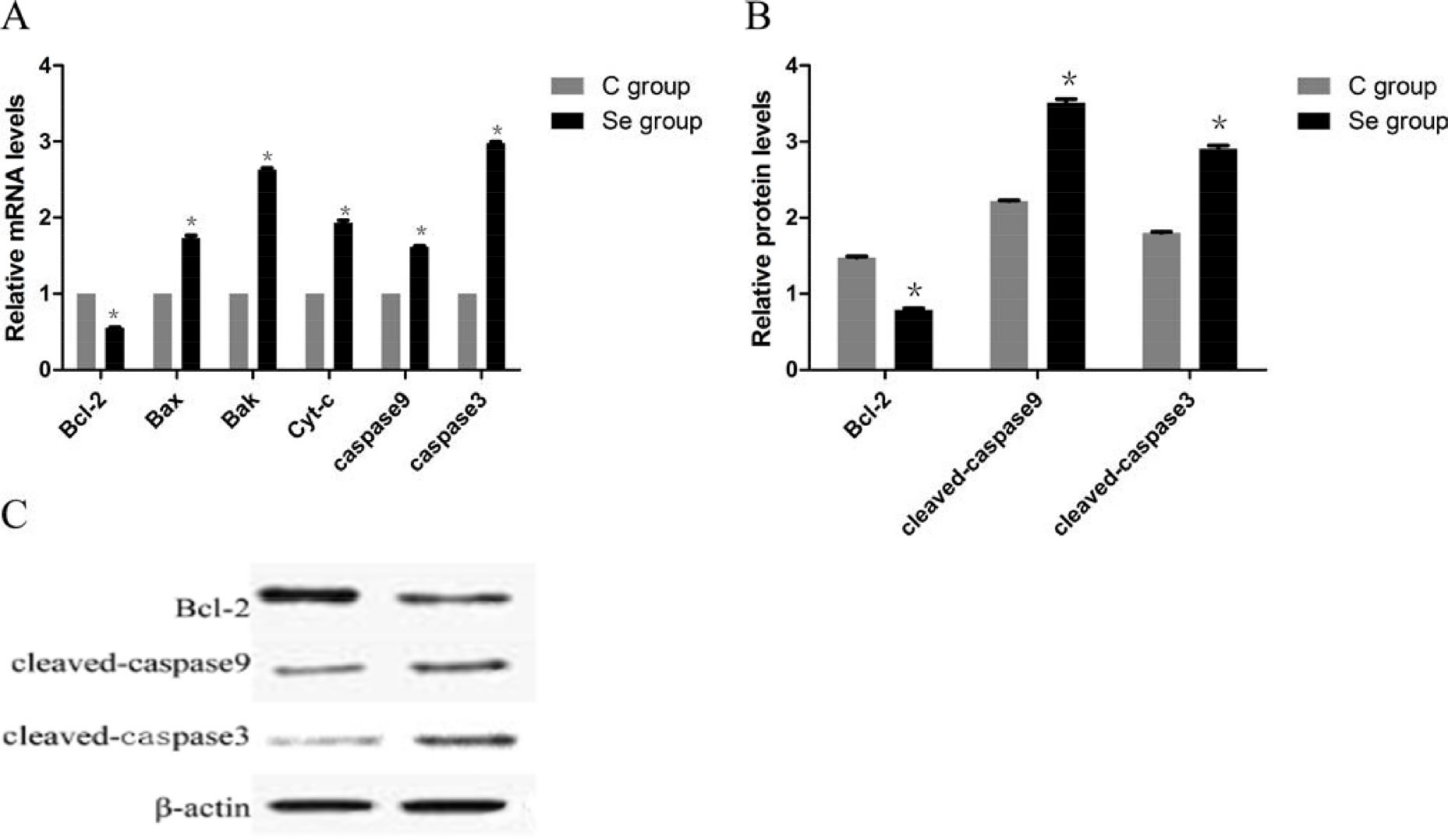
The effects of Se on apoptosis of oral squamous carcinoma cells (**A**) is the mRNA levels of apoptosis gens (Bcl-2, Bax, Bak, Cyt-c, Caspase9 and Caspase3). (**B**) is protein levels of apoptosis gens (Bcl-2, cleaved-caspase9 and cleaved-caspase3). (**C**) is the immunoblotting of Bcl-2, cleaved-caspase9, cleaved caspase3 and β-actin. Asterisk represents significant differences (*P* < 0.05) between control group and Se-treated groups. Each value represented the mean ± SD of five individuals.

To further investigate the effects of Se on apoptosis, we detected the mRNA and protein expressions of apoptotic genes (Bcl-2, Bax, Bak, Cyt-c, caspase9 and caspase3) by qRT-PCR and WB. As shown in Figure 5, both the mRMA and protein levels of anti-apoptotic gene (Bcl-2) were significantly decreased induced by Se. But the mRNA and protein levels of pro-apoptotic genes (Bax, Bak, Cyt-c, caspase9 and caspase3) showed significant increasing. Especially Caspase3 mRNA expression, it up-regulated about 2.98-fold. These results indicated that Se could induce apoptosis of oral squamous carcinoma cells.

## DISCUSSION

Cancer is one of the scenarios where too little apoptosis occurs, resulting in malignant cells that will not die [20]. Apoptosis could be used for treatment of cancer as it is a popular target of many treatment strategies [21–24]. Therefore, many anticancer drugs kill tumor cells by inducing apoptosis, this represents an important mechanism to exploit. OC is a multistage and slow progress, its initiation, proliferation, and development involved many mechanism and pathway, like apoptosis [25].Increased intake of Se may reduce the risk of degenerative diseases including cancer but excessive intake may be toxic. Previous study showed that Se induced apoptosis through elevating of ROS, mitochondrial dysfunction, cyt-c release, caspase3 activation, and DNA fragmentation [26]. The pathological sections showed that the oral cancer model of rat was successfully constructed by 4-NQO. In this study, we investigated that wether Na_2_SeO_3_ could improve the condition of OC via inhibiting the proliferative activity, promoting oxidative stress and cell apoptosis.

Se is beneficial synergistic effects on oxidative stability of tissues may result [27]. But, there are also many studies reported that Se could induced oxidative damage. For example, the increased lipid peroxidation and decreased antioxidant enzymes were caused by high concentrations of Se in coffee cell suspension cultures [28]. Hoffman et al. indicated that concentrations of over 100 ppm (mg/kg) organic Se could induced toxicity and oxidative stress in mallard ducklings [29]. In our results, the oxidative also was induced by Se via decreased activities of SOD and increased oxidative productions. However, Se have less effect on GSH-Px , this maybe resulted from that Se is functionally related as cofactor of GSH-Px. The occurrence of oxidative stress induced by Se may companied with up-regulated apoptosis [30].

Anti-apoptotic Bcl-2 promote cell survival by blocking programmed cell apoptosis through preventing Bax- and Bak-mediated mitochondrial apoptosis [31, 32]. In this work, we found the expression of Bcl-2 was decreased by Se treated accompany with the increased expressions of Bax and Bak in OC tissues of rat, suggesting that Se weakened the anti-apoptotic action of Bcl-2 and released more pro-apoptotic molecules like Bax and Bak, which were involved in pro-apoptotic process. Bax and Bak are regarded as key mediators for cyt-c release [33]. Cyt-c release from mitochondria, which then activates the caspase9. The activated caspase-9 in turn activates caspase-3 and initiates the proteolytic cascade to trigger apoptosis of OC cells by high concentration of Se [34, 35]. Our study was consisten with others, for instances, Uğuz et al. reported that high concentrations of Se could induced severe oxidative stress and caused cell death by activating modulating Ca^2+^ release, the caspases and cell apoptosis in human promyelocytic leukemia HL-60 cells [11]. the study of Chen et al. suggested that selenocystine, as a promising anticancer selenocompound, induces MCF-7 cell apoptosis by activating ROS-mediated mitochondrial pathway [36]. Therefore, our work suggested that apoptosis induced by Se may be one of the mechanisms of OC therapy.

## MATERIALS AND METHODS

### Animals

Forty adult Wistar rats (3 month-old) with an average weight of 200±20 g were purchased and were randomly divided into 4 groups, including contorl group (C group), low selenium group (L group), medium selenium group (M group) and high selenium group (H group). All animals were quarantined and acclimatized to laboratory conditions for 2 weeks. During the study, each rat was housed in a metal cage, with hardwood chips for bedding in an air-conditioned room under 12-h light/12-h dark cycles at a temperature of 22 ± 2°C. The method induced oral cancer by add 4-Nitroquinoline-1-oxide (4-NQO) (Sigma) at the concentration of 30ppm in their drinking water for 14 weeks. This step based on the method which described by Mehdipour et al and Abbasi et al. [7, 16]. At the same time, we were also intraperitoneally injected with Na_2_SeO_3_ (Sigma) 20, 30 and 40 μmol/kg, respectively. All the ethical and the humanity considerations were performed according to the Helsinki humanity research declaration during the experiments and the euthanasia of the animals. All the animals’ experiments were approved by the Ethics Committe.

### Cell cultrue

Rat oral squamous carcinoma cells were propagated in MEM medium supplemented with 10% FCS and antibiotics (penicillin 50 IU/ml and streptomycin 50 µg/ml) in a humidified atmosphere of 5% CO_2_ and 95% air at 37°C, then treated the OC cells by 0.5 mm Na_2_SeO_3_.

### Cell proliferation assays

The cells (5,000 cells/well) were seeded into 96-well plates, which were subsequently incubated for 24 h. The cells were then replenished with fresh complete medium containing selenium (dissolved in 0.1% DMSO), after which they were incubated for an additional 24 h. The cell proliferation was then evaluated by performing 3-(4,5-dimethylthiazol-2-yl)- 2,5-diphenyltetrazolium bromide (MTT) assays as described previously [17].

### Antioxidant enzyme analyses

Preparation of post-mitochondrial supernatant and determination of antioxidant enzyme activities and oxidative production levels were operated using the SOD, GSH-Px, dicarboxylic aldehyde (MDA) and reactive oxygen species (ROS) detection kits respectively (Nanjing Jiancheng Bioengineering Institute, P. R. China) according to their manufacturer’s protocol.

### RNA isolation and real-time quantitative PCR (qRT–PCR)

Total RNA was extracted using Trizol Reagent (Invitrogen) according to the manufacturer’s instructions. The concentration and purity of the total RNA were determined spectrophotometrically at 260 and 280 nm. The procedure of the reverse transcription was performed according to the manufacturer’s instructions (Invitrogen, Shanghai, China). Synthesized cDNA were stored at −20°C for PCR. Primer 5.0 Software was used to design specific primers for Bcl-2, Bax, Bak, cyt-c, caspase9, caspase3 and GADPH (a housekeeping gene used as an internal reference) based on known rat sequences (Table 1). Primers were synthesized by Huada Biotechnology Co. Ltd. Quantitative real-time PCR was performed on an ABI PRISM 7500 Detection System (Applied Biosystems, Foster City, CA).

**Table 1:** The sequences of primers for qRT–PCR

Genes’ name	Forward primer (5′–3′)	Reverse primer (5′–3′)	Product size (bp)
Bcl-2	GTGGGATACTGGAGATGAAGACCT	CAGCCAGGAGAAATGAAACA	550
Bax	GGCGAATTGGAGATGAACTG	GTCACTGTCTGCCATGTGGG	331
Bak	CAGCCCTGAGTTTGCGTAGAGAC	AGCGGATGAAAAGGGAGGATTGTA	417
cyt-c	CTTGGGCTAGAGAGCGGGA	GCTATTAGGTCTGCCCTTTCTCC	365
Caspase9	CTGAGCCAGATGCTGTCCCATA	GACACCATCCAAGGTCTCGATGTA	175
Caspase3	GAGACAGACAGTGGAACTGACGATG	GGCGCAAAGTGACTGGATGA	146
GAPDH	F:ACCACAGTCCATGCCATCAC	TCCACCACCCTGTTGCTGTA	453

The reaction conditions were as follows: 40 cycles of 95°C for 30 s, 95°C for 15 s and 60°C for 30 s, followed by 60°C for 30 s. Melting curve analysis demonstrated that each PCR product only exhibited one peak. The dissociation curves were analyzed using the Dissociation curve 1.0 Software (Applied Biosystems) for each PCR reaction to detect and eliminate any potential primerdimer formation and nonspecific amplification. The relative abundance of mRNA was calculated according to the method of -2^ΔΔCt^ [18], accounting for gene-specific efficiencies, and was normalized to the mean expression of GADPH.

### Extract protein and western blot (WB)

Protein extracts (20 μg/lane) were separated by sodium dodecyl sulfate polyacrylamide gel electrophoresis (SDS-PAGE) and transferred to an Immobilon-P polyvinylidene difluoride membrane (Millipore, Billerica, MA, USA). After blocking with SuperBlock T20 PBS blocking buffer (Thermo Fisher Scientific, Pittsburgh, PA, USA), the membranes were incubated with mouse monoclonal antibodies against Bcl-2 (1:800), caspase9 (1:1000) and caspase3 (1:1000), respectively. The bound antibodies were detected with 1:3000 diluted horse radish peroxidase (HRP)-conjugated secondary antibodies and visualized using Pierce ECL Western Blotting Substrate (Thermo Fisher Scientific), followed by exposing to film and digitally imaged. The relative intensity of each band was quantified by the software image J 1.43.

### Statistical analyses

The data were analyzed using SPSS No 16. One-Way Analysis Of Variance (ANOVA) was used to compare fold change difference [19]. The differences in mean values among groups were evaluated and the values were expressed as the means ± SD. The differences were considered to be significant if *P* < 0.05. All the statistical calculations were conducted using graphpad prism (version 5.0). Bars with small letters or “^*^” represented statistically significant differences (*P* < 0.05).

## CONCLUSIONS

In conclusion, the data show that excessive Se firstly induced intracellular oxidative stress, initiating programmed apoptosis and inhibiting proliferation of OC cells. Furthermore, the induction of apoptosis by administration Se suggests their potential as chemotherapeutic agents for many anticancer drugs function as anti-OC agents by inducing apoptosis.
